# The 90% effective concentration of alfentanil combined with 0.075% ropivacaine for epidural labor analgesia: a single-center, prospective, double-blind sequential allocation biased-coin design

**DOI:** 10.1007/s00540-024-03322-8

**Published:** 2024-03-05

**Authors:** Chang Jia, Bin Zou, Ying-Jie Sun, Bo Han, Yu-Gang Diao, Ya-Ting Li, Hui-Juan Cao

**Affiliations:** 1 Department of Anesthesiology, General Hospital of Northern Theater Command of the Chinese People’s Liberation Army, Shenyang, China; 2 Graduate School, Dalian Medical University, Dalian, China

**Keywords:** Alfentanil, EC90, Epidural analgesia, Labor analgesia

## Abstract

**Purpose:**

More literature studies have reported that alfentanil is safe and effective for labor analgesia. However, there is no unified consensus on the optimal dosage of alfentanil used for epidural analgesia. This study explored the concentration at 90% of minimum effective concentration (EC90) of alfentanil combined with 0.075% ropivacaine in patients undergoing epidural labor analgesia to infer reasonable drug compatibility and provide guidance for clinical practice.

**Methods:**

In this prospective, single-center, double-blind study, a total of 45 singleton term primiparas with vaginal delivery who volunteered for epidural labor analgesia were recruited. The first maternal was administered with 3 μg/mL alfentanil combined with 0.075% ropivacaine with the infusion of 10 mL of the mixture every 50 min at a background dose of 3 mL/h. In the absence of PCEA, a total of 15 mL of the mixture is injected per hour. The subsequent alfentanil concentration was determined on the block efficacy of the previous case, using an up-down sequential allocation with a bias-coin design. 30 min after epidural labor analgesia, the block of patient failed with visual analog score (VAS) > 3, the alfentanil concentration was increased in a 0.5 μg/mL gradient for the next patient, while the block was successful with VAS ≤ 3, the alfentanil concentration was remained or decreased in a gradient according to a randomized response list for the next patient. EC90 and 95% confidence interval were calculated by linear interpolation and prediction model with R statistical software.

**Results:**

In this study, the estimated EC90 of alfentanil was 3.85 μg/mL (95% confidence interval, 3.64–4.28 μg/mL).

**Conclusion:**

When combined with ropivacaine 0.075%, the EC90 of alfentanil for epidural labor analgesia is 3.85 μg/mL in patients undergoing labor analgesia.

## Introduction

Childbirth pain is one of the intense pains that women experience in their lifetime. Studies have shown that labor and immediate postpartum pains are associated with postpartum depression and traumatic stress symptoms, underscoring the importance of interventions for labor pain as well as postpartum pain [1]. Epidural analgesia is the most effective method of analgesia confirmed by the anesthesiologists and is the first choice for labor analgesia [2]. The guidelines recommend the use of low-concentration local anesthetics in combination with opioids, which not only reduces the rate of vaginal mechanical delivery but also provides effective analgesia [3]. Low-concentration ropivacaine in combination with sufentanil or fentanyl is commonly used [4, 5]. Alfentanil is a fast-acting μ-opioid receptor agonist, which has been indicated to have a rapid and accurate effect in the clinical application of epidural labor analgesia by many studies [6, 7]. But the effective concentrations of alfentanil used in these studies are different, and even high effective concentration are reported, which leads to a lack of reference for the use of clinical anesthetic drugs. The purpose of this study was to explore the concentration at 90% of minimum effective concentration (EC90) of alfentanil that could provide a satisfactory analgesic effect for primiparas in combination with 0.075% ropivacaine by a biased coin design up-and-down sequential method (BCD-UDM).

## Methods

### Selection and exclusion of subjects

This study was approved by the Medical Ethics Committee of the General Hospital of the Northern Theater of the Chinese People’s Liberation Army [No. Y (2022) 092] and registered with the China Clinical Trials Center (trial registration number: ChiCTR2300067317). The trial was officially conducted in the Obstetrics and Delivery Center in accordance with the Declaration of Helsinki, and a total of 45 parturients who met the criteria were eventually enrolled for clinical observation. All parturients and their families signed an informed consent form. The inclusion criteria were as follows: (1) singleton, fetal head position and full term (37–42 weeks of gestation), (2) age 20–35 years, height 150–170 cm, body mass index (BMI) < 35 kg/m^2^, American Society of Anesthesiologists (ASA) Class II, (3) uterine orifice dilation of 2–3 cm, regular uterine contractions assessed by the obstetrician and midwife, (4) eligible for vaginal delivery. The maternal with any of the following conditions during the trial would be excluded and discontinued from observation: (1) unable to cooperate with the epidural puncture and visual analog score (VAS) evaluation, (2) VAS < 6 before analgesia, (3) converted to cesarean delivery for various reasons during labor analgesia, (4) allergic to local anesthetics or opioids, (5) contraindications to intervertebral block, (6) pregnancy complications such as eclampsia and gestational diabetes mellitus or other serious organic diseases, (7) rapid cervical orifice change (cervical orifice dilation ≥ 5 cm) within 45 min after epidural analgesia, (8) withdrawal midway and those with suspicious results.

### Procedure and method of anesthesia

When the cervical orifice was enlarged to 2–3 cm, the parturient was placed in the delivery room, and connected to a maternal fetal monitor (SRF618K9, Guangzhou Sunray Medical Apparatus, Guangzhou, China). The electrocardiogram (ECG) was monitored and blood pressure (BP), heart rate (HR), pulse oxygen saturation (SPO_2_), fetal heart rate (FHR), uterine pressure and temperature were recorded. Upper limb venous access was opened, and sodium lactate Ringer’s solution was given for fluid replacement. The parturient was instructed to a left-lying position and an epidural puncture was performed in the L2-3 space. The catheter was placed into the epidural space with 4 cm towards the head, and 3 mL of 1% lidocaine was given as a test dose, then the catheter was properly secured after the test dose was safe. The maternal was excluded if the dura was punctured or the epidural tube was failed to insert. An epidural catheter was connected to an electronic analgesic pump and programmed intermittent epidural injection (PIEB) combined with patient-controlled epidural analgesia (PCEA) mode was selected. According to the guidelines [3] and clinical experience, when 10 mL of the study solution was given as the pulse volume in this experiment, the pulse interval was set to 50 min to minimize the effect of maternal motor block, and the other parameters were set to 3 mL of background dose, 8 mL of maternal self-control dose, and 20 min of lockout time.

The VAS evaluation (0 for no pain, 10 for intolerable pain) and leg lift test were performed. The parturients were asked to raise both legs at a 45° angle and kept straight for 4 s while closing the eyes. The latest Bromage score (1—complete motor block; 2—almost complete motor block, patient can only move the foot; 3—partial motor block, patient has knee motion; 4—detectable hip flexion and extension weakness, patient can lift the legs but cannot maintain them; 5—no hip flexion and extension weakness, patient can lift the legs for more than 10 s; 6—no weakness) was used to assess the degree of motor block and motor intensity in the lower extremity. The degree of upper sensory block was assessed by the movement of an alcohol-impregnated swab from the level of S2 toward the head. In the lower extremity, the plane of block was assessed by stimulation of the inguinal fold (L1), anterior thigh (L2), medial knee (L3), medial malleolus (L4), dorsoventral area between the big toe and second toe (L5), lateral heel (S1), and medial popliteal fossa (S2). All of the above indicators were recorded at 15, 30, and 45 min after epidural analgesia and fully dilation of cervical orifice.

Adverse events during labor analgesia were addressed promptly. In case of hypotension (systolic blood pressure dropping more than 20% of the basal value or less than 90 mmHg), the parturient was treated in lateral position or with intravenous fluids, and norepinephrine or ephedrine could be given if there was no significant relief. The maternal bradycardia (HR < 50 bpm) was treated with atropine. When the fetal bradycardia (FHR < 110 bpm for more than 10 min) occurred, the parturient was examined and evaluated by the obstetrician and midwife. If there was no significant improvement after changing the position or administering oxygen via high-flow nasal cannula, emergency cesarean delivery was performed to ensure fetal life. The VAS 30 min after epidural labor analgesia less than or equal to 3 was considered effective. In cases of incomplete analgesia, the possible causes were considered and appropriate measures were taken according to the manifestations of incomplete analgesia. For example, if the analgesic plane was adequate (T10-S4) but the analgesic intensity was insufficient, the concentration of analgesic could be increased. For bilateral blocks and insufficient analgesic planes, a large volume (5–15 mL) of low-concentration local anesthetic was used for planar diffusion. If maternal labor pain was relieved, the next patient would receive a graded increase in alfentanil concentration. Otherwise, the remedy was considered ineffective and marked as suspicious, the case was excluded from the study, and the next woman also adapted this alfentanil concentration.

### Type and methodology of the study

The type of this study was single-center, prospective, and double blind. Epidural puncture and catheterization, preparation of analgesic solution and assessment of analgesic effect were performed by anesthesiologists who were unaware of the study protocol. The study used BCD-UDM, which was the optimal upper and lower design in the general category, allowed accurate estimation of target doses for low or high quantiles, and had the advantages of small sample size and reasonably simple study performance. Based on the results of the pretest, the epidural concentration of alfentanil was set at 3 μg/mL in the first parturient, and then the concentration in the next parturient was depended on the block effect in the previous parturient. If the block failed (VAS 30 min after epidural labor analgesia more than 3 was considered incomplete analgesia), the concentration of alfentanil was increased in a gradient of 0.5 μg/mL for the next patient. If the block was successful (VAS 30 min after epidural labor analgesia less than or equal to 3 was considered effective analgesia), there was an 11% (*b* = 0.11) probability of a one-unit decrease (0.5 μg/mL) and an 89% (1− *b* = 0.89) probability of no change for the next parturient according to the BCD-UDM method. The sequential assignment of the biased coins was achieved by a computer-generated list of random responses, which was prepared by a biostatistician with excel. This random list was used by a research assistant to develop analgesic protocol for the next parturient based on the observer-recorded response of the previous parturient. The protocol was delivered in a closed envelope to the researcher who prepared the analgesic solution.

### Primary and secondary outcomes

The VAS 30 min after epidural analgesia was used as the primary observation, and the primary outcomes were EC90 and 95% confidence intervals (CIs) for alfentanil. Secondary outcomes were recoded, including the values of the mentioned indicator at five time points, duration of analgesia and labor, mode of delivery, number of PCEA, oxytocin dosage after analgesia and amount of epidural analgesic solution. Midwifes measured Apgar scores at 1 and 5 min after birth and withdraw cord artery blood for pH measurement. The incidence of lower limb numbness, skin pruritus, urinary retention, nausea, vomiting and other adverse effects were observed and recorded. Finally, maternal satisfaction was scored.

### Statistical analysis and sample size calculation

Based on previous studies, a minimum of 20–40 subjects was required to obtain a stable estimated sample size [8–10], so 45 subjects were ultimately included in this study. The inherent expected failure rate according to the biased coin design sequential method was 11%, so the study endpoint for this experiment was at least 41 cases with successful block. The equivariate regression estimator for EC90 was a linear interpolation of the dose between p*(*r*) and p*(*r* + 1): EC90 = (*x*(*r* + 1) − *x*(*r*)) + *x*(*r*).Where *x* (*r*) = maximum (*x* (1): p*(i) ≤ 0.9), p* (i) was the main influence dose for *x*(*i*) supervision, *i* = 1, 2, 3,…, *k*, which was estimated by the Pool Adjacent Offender Algorithm (PAVA). The observed rate of *p* = (*p*(1), *p*(2),. *p*(*k*)) might not increase with increasing dose level, which was an implicit assumption of dose seeking studies. Therefore, the PAVA algorithm was first used for equivalence regression to obtain increasing adjustment rates based on *p*' = [p*(1) ≤ p*(2) ≤ *p*(*k*)]. Based on these results, the EC90 of alfentanil was calculated. Considering the independent linear relationship between doses, the 95% CI of EC90 was obtained according to the prediction model [*E*(*Y*∣*X*) = *β*0 + *β*1*X*].

## Results

### Recruitment and demographic characteristics of subjects

**Fig. 1:**
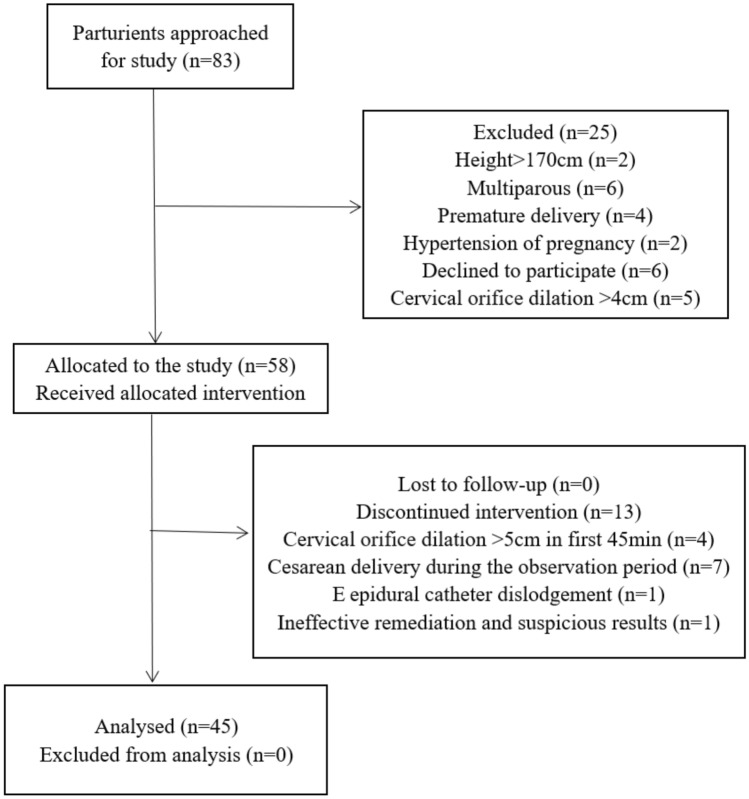
Recruitment of subjects

In this study, 83 women who received labor analgesia at our hospital from January 5 to February 28, 2023, were followed up. Twenty-five women were excluded from the study due to height over 170 cm (*n* = 2), multiple pregnancy (*n* = 6), preterm delivery (*n* = 4), gestational hypertension (*n* = 2), cervical orifice dilatation greater than 4 cm (*n* = 5), and refusal to sign an informed consent form (*n* = 6). Of the 58 women who met the inclusion criteria, 4 women’s cervical orifice dilated greater than 5 cm within 45 min, 7 women were converted to cesarean section for various reasons, 1 case had epidural catheter dislodgement, and 1 case had ineffective remediation and suspicious results, so these parturients were excluded. Finally, 45 women completed clinical observation, as shown in Fig. 1. Maternal height, age, weight and other demographic characteristics are recorded in Table 1.

**Table 1:** Maternal demographic characteristics

Characteristics	Finding (*N* = 45)
Height, cm	163.7 ± 4.3
Weight, kg	70.7 ± 8.8
BMI, kg/m^2^	26.3 ± 3.0
Age, years	28.8 ± 2.6
Gestational age, weeks	39.3 ± 0.7
Hemoglobin, g/L	122.5 ± 11.2
Blood platelet, 10^9^/L	197.8 ± 42.2
Hematocrit, %	37.3 ± 3.1

Data are presented as mean ± standard deviation

*BMI* body mass index

### Indicators at different time points

**Fig. 2:**
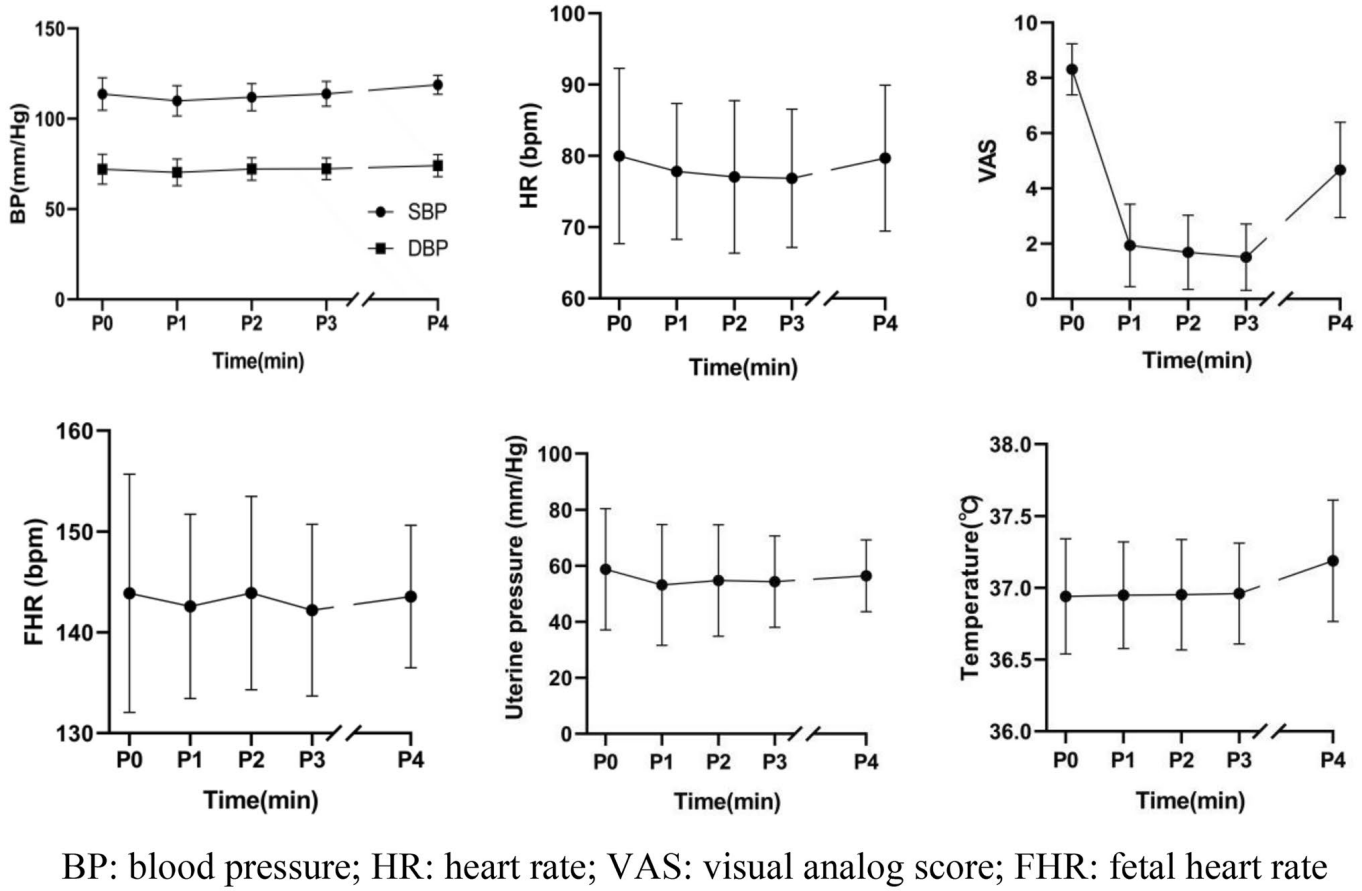
Line plot of mean and standard deviation. *BP* blood pressure, *HR* heart rate, *VAS* visual analog score, *FHR* fetal heart rate

In Table 2, various indicators were recorded before epidural analgesia (P0), 15 min (P1), 30 min (P2), 45 min (P3) after epidural analgesia and at complete opening of the cervical orifice (P4). All women had no motor block. The median sensory level block plane at P1–P3 was T10 with an interquartile range of T9–11, whereas the sensory level at P4 was decreased to T11 with an interquartile range of T9–11. The median sensory block plane in the lower extremity was L3 at the P1 time point and L1 at the P2–P4 time point, the interquartile range was L1–5 at the P1–P3 time point and L1–1 at the P4 time point. Compared with the pre-analgesic period, the VAS scores were significantly lower after analgesia, and slightly higher after total cervical orifice opening. The VAS scores after total cervical orifice opening were higher than those within 45 min after analgesia, but still slightly lower than those before analgesia. The body temperature at P4 time points was slightly higher than that at other time points. To more visually reflect the changes of each index at different points, the results of SBP, DBP, HR, FHR, uterine pressure, VAS score and body temperature were plotted as line graphs, as shown in Fig. 2.

**Table 2:** Observed indicators at different time points

Indicators	Finding (*N* = 45)				
	P0	P1	P2	P3	P4
SBP, mmHg	113.7 ± 8.9	109.9 ± 8.3	111.9 ± 7.4	113.9 ± 6.8	118.8 ± 5.2
DBP, mmHg	72.1 ± 8.2	70.3 ± 7.3	72.1 ± 6.2	72.3 ± 6.0	74.1 ± 6.1
HR, bpm	80.0 ± 12.2	77.8 ± 9.4	77.0 ± 10.6	76.8 ± 9.6	79.7 ± 10.1
SPO_2_, %	98.0 ± 1.2	98.0 ± 1.0	98.0 ± 1.6	98.1 ± 1.0	98.2 ± 0.9
FHR, bpm	143.9 ± 11.7	142.6 ± 9.1	143.9 ± 9.5	142.2 ± 8.4	143.6 ± 7.0
Uterine pressure, mmHg	58.4 ± 21.6	53.2 ± 21.4	54.8 ± 19.7	54.4 ± 16.2	56.4 ± 12.7
VAS score	8.3 ± 0.9	1.9 ± 1.5	1.7 ± 1.3	1.5 ± 1.2	4.7 ± 1.7
Temperature, ℃	36.9 ± 0.4	36.9 ± 0.4	36.9 ± 0.4	37.0 ± 0.3	37.2 ± 0.4
Bromage score	–	6 [6–6]	6 [6–6]	6 [6–6]	6 [6–6]
Upper sensory level	–	T10 [9–11]	T10 [9–11]	T10 [9–11]	T11 [9–11]
Lower extremity block plane	–	L3 [1–5]	L1 [1–5]	L1 [1–5]	L1 [1–1]

Data are presented as mean ± standard deviation or median [interquartile range]. P0 = before epidural analgesia; P1 = 15 min after epidural analgesia; P2 = 30 min after epidural analgesia; P3 = 45 min after epidural analgesia; P4 = at complete opening of the cervical orifice

*SBP* systolic arterial pressure, *DBP* diastolic arterial pressure, *HR* heart rate, *SPO*_2_ pulse oxygen saturation, *FHR* fetal heart rate, *VAS* visual analog scale

### EC90 of alfentanil

**Fig. 3:**
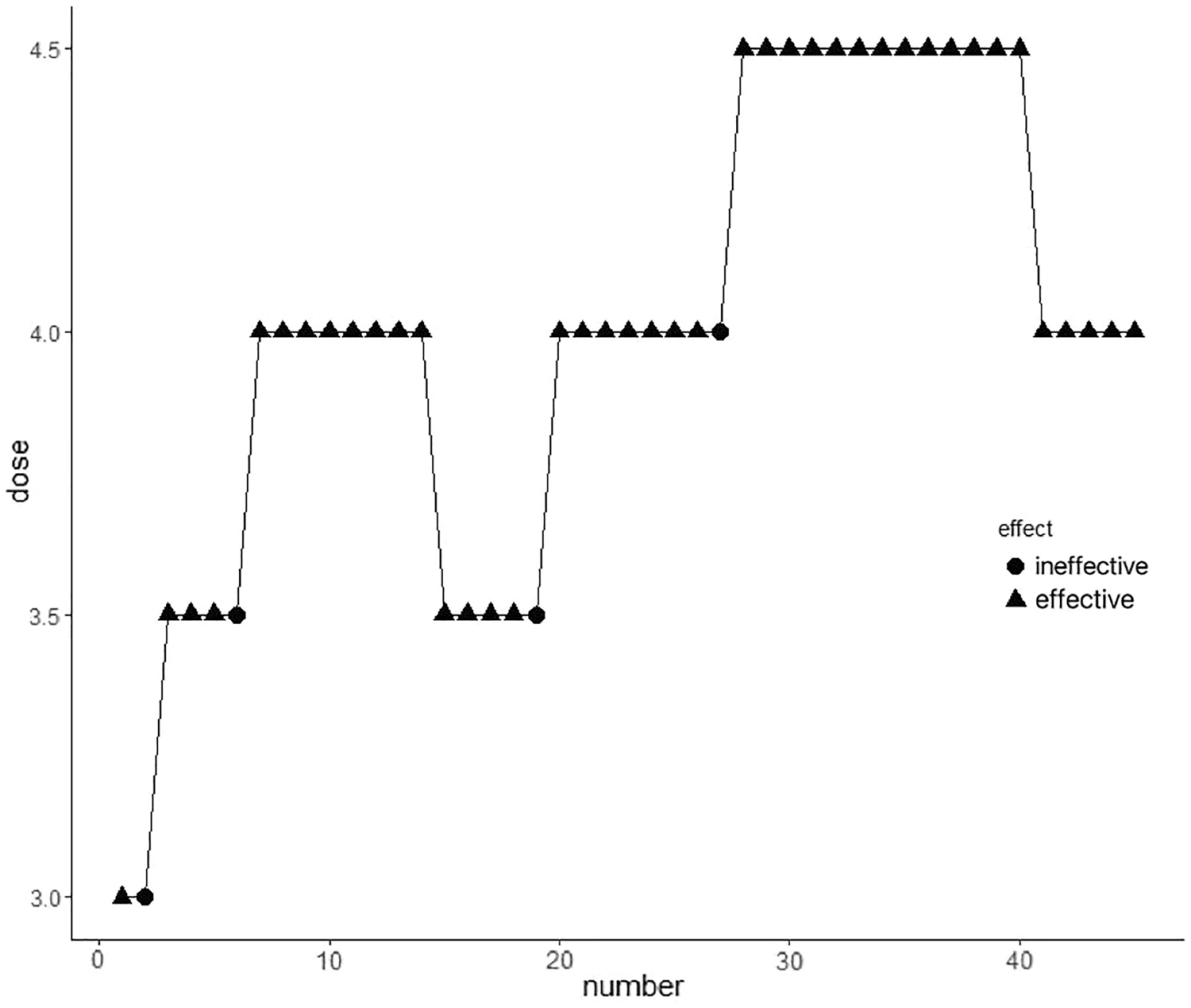
Effective and ineffective reaction sequences

The effective and ineffective response sequences of 45 consecutive parturients, who were given epidural different concentrations of alfentanil combined with 0.075% ropivacaine are shown in Fig. 3. The response rates for four concentrations of alfentanil at 3, 3.5, 4 and 4.5 μg/mL are shown in Table 3. The EC90 of alfentanil was 3.85 μg/mL (95% CI 3.65–4.28) by isotonic regression, and most parturients could achieve satisfactory blockade with epidural administration of 4 μg/mL alfentanil combined with 0.075% ropivacaine, where only one out of 21 parturients had poor analgesic effect.

**Table 3:** Response rate of 45 parturients

Dose of alfentanil	Cases with successful analgesic effect	Cases	Response rate (observed)	Response rate (PAVA)
3.0	1	2	0.5	0.5
3.5	7	9	0.78	0.78
4.0	20	21	0.95	0.95
4.5	13	13	1	1

*PAVA* Pool Adjacent Offender Algorithm

### Maternal and neonatal outcomes

The maternal outcomes, including the duration of labor and analgesia, oxytocin dosage and amount of epidural analgesic solution are recorded in Table 4. There were seven women used PCEA and four of them received twice. In addition, 2 parturients developed slight pruritus in the chest, which gradually disappeared after about half an hour. There was no hypotension, bradycardia, respiratory depression, nausea, vomiting and other symptoms in the 45 parturients. There were 14 cases of lateral episiotomy and 31 cases of natural delivery without forceps-assisted delivery. The mean maternal satisfaction score was above 9. The neonatal outcomes are recorded in Table 5. The Apgar scores at 1 and 5 min was 10 in all newborns, and their pH of umbilical artery blood was 7.28 ± 0.05.

**Table 4:** Maternal outcomes

Outcomes	Finding (*N* = 45)
Labor stage 1, min	420.9 ± 161.1
Labor stage 2, min	60.0 ± 27.5
Labor stage 3, min	9.0 ± 3.7
Duration of epidural analgesia, min	311.6 ± 155.4
Oxytocin dosage after analgesia, u	2.6 ± 1.7
Amount of epidural analgesic solution used, ml	84.2 ± 35.3
The number of PCEA	7 (16)
Lateral episiotomy	14 (31)
Natural labor	31 (69)
Forceps delivery	0 (0)
Nausea	0 (0)
Vomiting	0 (0)
Pruritus	2 (4)
Respiratory depression	0 (0)
Hypotension	0 (0)
Bradycardia	0 (0)
Maternal satisfaction	9.7 ± 0.7

Data are presented as mean ± standard deviation or number (percentage)

*PCEA* patient-controlled epidural analgesia

**Table 5:** Neonatal outcomes

Outcomes	Finding (*N* = 45)
Birth weight, g	3275.3 ± 357.7
Apgar scores at 1 min	10 [10–10]
Apgar scores at 5 min	10 [10–10]
UA blood pH	7.29 ± 0.05

Data are presented as mean ± standard deviation or median [interquartile range]

*UA* umbilical artery

## Discussion

Significant placental transfer can limit the dose of opioids when fentanyl, alfentanil or sufentanil is administered intravenously, epidurally or intrathecally [11]. In this consecutive dose study, the EC90 for alfentanil combined with 0.075% ropivacaine for epidural labor analgesia was found to be 3.85 μg/ml (95% CI 3.64–4.28), implying that 4 μg/mL alfentanil could provide a satisfactory analgesia for most women. A study by Hill [12] showed that the minimum effective dose of alfentanil combined with 0.125% bupivacaine was 266 μg/h, which was higher than the concentration of alfentanil in our study. The study by Bader [7] showed that 0.125% bupivacaine combined with 5 μg/mL alfentanil in the clinical application of epidural labor analgesia had a rapid onset of action, definite effect and high patient satisfaction, but the concentrations of both local anesthetic and alfentanil were slightly higher than those in our study. Hallidy et al. [13] showed that ultra-low concentration (≤ 0.08%) local anesthetic increased the likelihood of spontaneous vaginal delivery, reduced motor block and shortened the duration of the second stage of labor compared to high concentration local anesthetic. Therefore, in our study, 0.075% ropivacaine was used and none of the women experienced motor block.

In our clinical investigation, we endeavored to identify the optimal initial concentration for the combined administration of alfentanil and 0.075% ropivacaine in epidural labor analgesia. This was assessed in a cohort of 15 females. Our objective was to evaluate both the therapeutic and adverse effects of varied concentrations of alfentanil, in conjunction with 0.075% ropivacaine. In general, the efficacy and safety of local anesthetic concentrations are determined by their specific formulation. Research has shown that a combination of 0.075% ropivacaine and bupivacaine with 2 μg/mL fentanyl is equally effective for patient-controlled epidural analgesia in relieving labor pain [14]. Referencing studies such as those by Bader et al. [7], we hypothesized that the effective concentration 90% (EC90) for alfentanil in this combination falls within the range of 0.5–5.0 μg/mL. Our methodology aligned with the principles of the Observed Adverse Effect Level (OAEL) and the Minimum Anticipated Biological Effect Level (MABEL) for determining the initial dose. The preliminary phase of our study commenced with an alfentanil concentration of 0.5 μg/mL, incrementally increasing in 0.5 μg/mL intervals. The mean Visual Analogue Scale (VAS) scores for pain in five females, at alfentanil concentrations ranging from 0.5 to 2.5 μg/mL, were 6.4 at 15 min and 4.8 at 30 min post-administration, indicative of visceral pain. Post-lidocaine administration, significant analgesia was observed. Considering potential insufficiency in alfentanil concentration as a cause for analgesic block failure, we incrementally augmented the alfentanil concentration in the analgesic solution. At an alfentanil concentration of 3 μg/mL, notable improvement was observed: one patient exhibited a maternal VAS score of 3 at 30 min of epidural analgesia without adverse effects, while another at the same concentration had a VAS score of 4.2 at 30 min following the administration. The average VAS score for these two patients at 15 min post-administration was 4.5. Subsequently, the analgesic efficacy and adverse reactions were monitored and recorded in eight cases (two per concentration level) with alfentanil concentrations ranging from 3.5 to 5 μg/mL. Notably, a patient with a concentration of 3.5 μg/mL had a VAS score of 4.1 at 30 min post-administration. The 15 min and 30 min VAS scores for females at concentrations of 4, 4.5 and 5 μg/mL were below 3, with no adverse maternal reactions observed. Following childbirth, umbilical artery blood samples were collected for analysis, and the Apgar scores of the neonates were within normal ranges at 1–5 min, suggesting no adverse neonatal effects. Therefore, the alfentanil concentration of 3 μg/mL was finally identified as the initial concentration for this analgesic regimen.

Many studies have found that larger doses of opioids are associated with a higher incidence of side effects, such as pruritus, drowsiness, fever, nausea and vomiting [15–17]. In this study, only 2 cases of parturient experienced slight pruritus in the chest for a short time. In a study of 0.1% ropivacaine combined with 0.5 μg/mL sufentanil, the incidence of pruritus was 14.3% [18]. Moreover, 5 women had obvious fever when the uterine orifice was completely dilated with the progress of labor. However, the body temperature of these women was higher than 37.3 ℃ when entering the delivery room, and the mechanism of epidural-related maternal fever will also be further explored. In addition, there were no adverse reactions such as nausea, vomiting or drowsiness. So the choice of appropriate opioid concentrations can avoid the adverse effects caused by opioid overdose.

The pharmacokinetic studies have shown that alfentanil has a faster distribution rate in the epidural space than fentanyl and sufentanil, resulting in a faster analgesic effect [19]. Nevo et al. [20] showed that the onset of epidural labor analgesia was significantly correlated with pain scores after 60 min, and longer analgesic onset, higher VAS at 60 and 120 min. In this study, there was no significant change in the uterine pressure at different time points. The VAS of the women with successful block were lower than 4 in the first 45 min after epidural analgesia, and did not vary much in longer time, indicating that alfentanil has a fast onset time, good and stable analgesic effect. The nature of labor pain changed with the progress of labor, and visceral pain in the first stage of labor changed to somatic pain in the second stage of labor during the uterine orifice completely dilation. At this time, the VAS increased. In this study, the VAS of 7 parturients was higher than 6 and had one PCEA. 4 of them failed to block 30 min after epidural analgesia, then another PCEA was also done to relieve labor pain. The average maternal satisfaction score was above 9. In our observation, it was found that the effect of controlled analgesia in the first stage of labor was better than that in the second stage of labor. We speculate that the somatic pain in the second stage of labor can be relieved by increasing the concentration of local anesthetics, but which need to be verified by further studies. Meanwhile, we look forward to the emergence of new products of electronic analgesia pump that can adjust the concentration of local anesthetics or opioids when necessary, to reduce the maternal breakthrough pain and realize individualized analgesia. In conclusion, it is recommended to use 4 μg/ml alfentanil combined with 0.075% ropivacaine combined with for epidural labor analgesia, which has a perfect and rapid analgesic effect with few adverse effects.

There are some limitations of the present study. First, the results of this study were only applicable to the strict conditions of this experiment, such as the fixed concentration of 0.075% ropivacaine, as well as the fixed time and dose settings of the electronic analgesic pump, which undoubtedly hindered the generalization of the results. Second, the rapid progress of labor is a common factor of breakthrough pain [21–23], the evaluation of labor before analgesia is important. It has been shown that PCEA + PIEB protocol is more advantageous in reducing breakthrough pain [24]. This study only used a set analgesic mode with pulse interval of 50 min, pulse of 10 ml and self-control of 8 ml, so other intervals, volumes and self-control volumes still need further research. Finally, narrowing the increasing and decreasing gradient of alfentanil concentration is expected to provide a more accurate EC90 and a smaller 95% CI range, which can better guide the use of alfentanil in labor analgesia.

## Conclusion

In this study, alfentanil was combined with 0.075% ropivacaine during epidural labor analgesia, and the EC90 of alfentanil was 3.85 μg/mL, with a 95% CI of 3.64–4.28 μg/mL. During the analgesic process, the maternal pain relief was significant, the movement was not restricted, and the maternal satisfaction was high. In addition, there was no abnormality in the fetal heart rate during labor and the newborn was born in good condition. However, the effective concentration of alfentanil in other analgesic modes when combined with 0.075% ropivacaine still needs further clinical observation and exploration.

## Data Availability

All data in this study are from the Clinical Trial Management Public Platform (trial registration number: ChiCTR2300067317). You can obtain them through the following website: http://www.medresman.org.cn/. If data are not available from the website, data supporting the findings of this study can be obtained from the corresponding author.
